# Application of a novel microscopic technique for quantifying CA125 binding to circulating mononuclear cells in longitudinal specimens during treatment for ovarian cancer

**DOI:** 10.1186/s13048-022-00957-7

**Published:** 2022-02-26

**Authors:** Kornél Lakatos, Germán González, Jawad Hoballah, Jeff Brooker, Sinyoung Jeong, Conor Evans, Petra Krauledat, W. Peter Hansen, Kevin M. Elias, Manish Patankar, Vilmos Fülöp, Panagiotis A. Konstantinopoulos, Daniel W. Cramer

**Affiliations:** 1 Gynecologic Oncology Laboratory, Brigham and Women’s Hospital, 75 Francis Street, Boston, MA 02132 USA; 2 PNP Research Corporation, LLC, 282 S COUNTY RD, Drury, MA 01343 USA; 3 Thorlabs Imaging Systems, 108 Powers Ct, Sterling, VA 20166 USA; 4 Wellman Center for Photomedicine, Massachusetts General Hospital, Harvard Medical School, 55 Fruit Street, Boston, MA 20114 USA; 5 Department of Obstetrics, Gynecology and Reproductive Biology, Harvard Medical School, Boston, MA USA; 6 Dana-Farber Cancer Institute, 75 Francis Street, Boston, MA 02115 USA; 7 Department of Obstetrics and Gynecology, University of Wisconsin-Madison, Madison, WI USA; 8 Department of Obstetrics and Gynecology, Military Hospital Medical Centre, Hungarian Defense Forces, Budapest, Hungary; 9 Faculty of Healthcare, University of Miskolc, Miskolc, Hungary; 10 Semmelweis University, Budapest, Hungary; 11 Clinical Medicine Doctoral School, University of Szeged, Szeged, Hungary; 12 Obstetrics and Gynecology Epidemiology Center, Brigham and Women’s Hospital, 221 Longwood Avenue, Boston, MA 02115 USA

**Keywords:** Ovarian Cancer, CA125, Mononuclear cells, Nanoparticles, Microscopy

## Abstract

**Background:**

Measurement of serum CA125, an antigenic fragment of human mucin 16 (MUC16), is used to monitor the clinical progression of epithelial ovarian cancer (EOC). However, rather than simply a passive marker reflecting tumor burden, MUC16 may have a more active role by binding to immune cells and altering their tumor response. We developed a research tool to measure MUC16-binding to the surfaces of peripheral blood mononuclear cell (PBMC) subtypes and tested its research value using specimens collected serially from a woman being treated for high grade serous EOC.

**Methods:**

Cryopreserved PBMCs were mixed with anti-CA125 antibody-labeled plasmonic gold nanoparticles (PNPs) to detect cell surface MUC16-binding along with fluorescent stains to identify B cells, NK cells, NK-T cells, T cells, and monocytes. From 3D darkfield images, a computer algorithm was applied to enumerate PNP-binding and fluorescence microscopy to identify cell lineage. Average MUC16-binding was determined by fitting a Poisson distribution to PNP-counts across similar cell types. MUC16-binding to cell types was correlated with treatment details, CA125 levels, and complete blood count (CBC) data.

**Results:**

Over a 21-month period, monocytes had the highest level of MUC16-binding which was positively correlated with serum CA125 and inversely correlated with circulating monocyte and lymphocyte counts. Fluctuations of PNP-binding to NK cells were associated temporally with types of chemotherapy and surgical events. Levels of MUC16 bound to NK cells were positively correlated with levels of MUC16 bound to T and NK-T cells and inversely correlated with circulating platelets.

**Conclusions:**

Assessment of MUC16-binding among cryopreserved PBMC cell types can be accomplished using darkfield and fluorescence microscopy. Correlations observed between level of binding by cell type with serum CA125, CBC data, and treatment details suggest that the new techniques may offer novel insights into EOC’s clinical course.

## Introduction

In the search for a reliable biomarker for epithelial ovarian cancer (EOC), mice were immunized with an EOC cell line and splenocytes harvested to look for cancer-specific immune responses. This effort led to creation of a monoclonal antibody that reacted with sera from most women with EOC [1, 2]. The antibody was called OC125 and the antigen it detected, CA125. CA125 was found to be part of a larger glycoprotein eventually identified as a member of the mucin family (MUC16) [3]. Higher CA125 levels at presentation of EOC predict poorer survival and serial measurements can be used to predict recurrence [4]. While this could simply reflect greater or increasing tumor burden, observations suggest that MUC16 has a more active role in disease progression by binding to cell surfaces of peripheral blood mononuclear cells (PBMCs) including NK cells and blunting their tumoricidal ability [5–7]. These experiments relied upon fluorescent labels with conventional flow cytometry which may be limited in detecting low levels of cell surface antigens due to auto-fluorescence.

These limitations have been addressed in a series of papers from our group describing technology that opens a new window for assessing the degree of MUC16-binding to PBMCs. This technology involves: 1) plasmonic gold nanoparticles (PNPs) conjugated to anti-CA125 antibodies as optical nanoprobes; 2) high contrast darkfield microscopy to detect PNPs bound to PBMCs and 3) a computational algorithm for automated counting of the bound nanoparticles. PNPs have a unique property called plasmonic resonance, which causes them to strongly scatter specific wavelengths of light to enable single nanoparticle detection. Data were presented from women with EOC showing that their PBMCs (collected before therapy) had greater levels of MUC16 bound to their cell surfaces compared to PBMCs from healthy women [8]. In subsequent work, we improved the technique by adding fluorescence capacities to the microscope, enabling analyses on subsets of PBMCs [9, 10]. EOC cancer patients exhibited higher levels of MUC16 bound to T cells, NK cells and B cells compared to healthy controls [10].

In the study presented here, we now apply the refined technology to longitudinal specimens collected serially from a single patient with advanced serous EOC over a 21-month period of her care. We correlated levels of binding by cell type with serum CA125, CBC data, and treatment details which, we believe, suggest that the new technology can offer novel insights into EOC’s clinical course.

## Materials and methods

### Clinical specimens

In 2016, we began collecting blood to study MUC16-binding to PBMCs in women with presumed ovarian cancer prior to any therapy and later from healthy women. A patient who had been diagnosed with stage IIIC high-grade serous ovarian knew about the protocol and requested her blood be studied. Diagnosed in 2012, she had undergone suboptimal debulking followed by carboplatin and Taxol chemotherapy. Over the next 8 years, she required additional surgery, additional chemotherapy, and trials of novel therapies. The focus in this paper is the period of her care between 8/2017 and 4/2019. This period is of interest because: the sampling was frequent; there was clinical evidence that the patient’s tumor burden was increasing; and multiple therapies were tried. When clinical specimens were obtained, an extra calcium EDTA blood tube was drawn for the research and processed by the same laboratory to preserve viable PBMC according to the protocol described [8]. Briefly PBMC were isolated by Ficoll-density gradient centrifugation, washed in phosphate buffered saline (PBS), re-suspended in complete cell culture medium, and the cell concentration determined. The PBMC were then re-centrifuged and re-dispersed in a mixture of culture media and DMSO. Aliquots of 0.1 ml with cell concentrations of ten million per ml. were created and stored at -80 ^0^C prior to transfer to liquid nitrogen for shipment to PNP facilities in Sterling, VA where the specimens were analyzed.

Preparing specimens and analysis of MUC16 binding to PBMCs.

The process of preparing the specimens for microscopic analysis is similar to that described by González et al. [10]. Briefly, the PBMCs were thawed and stained separately, first with fluorescent antibodies to detect specific subsets of PBMCs and second with gold nanoparticle conjugated with anti-CA125 antibodies to detect cell-surface MUC16. Fluorescent labels for phenotyping T cells, B cells, NK cells and monocytes were obtained from Biolegend (San Diego, CA, USA) and included: BV510 anti-human CD45, BV421 anti-human CD19, AF488 anti-human CD14, PE anti-human CD56(NCAM), and AF647 anti-human CD3 antibody with catalog numbers, 368526, 302234, 325610, 318306, and 300416, respectively. Gold nanoparticles, eighty nanometers in size, conjugated with anti-CA125 antibody were purchased from PNP Research Corporation (Drury, MA, USA, custom conjugation). Gold staining was performed by incubating the sample with gold reagents at a ratio of 10^4^ PNPs per cell in 500 uLof PBS with 5% BSA, for five minutes, under continuous mixing conditions. After bound-free nanoparticle separation, samples were reconstituted to a cell density of 25 M cells/ml, in PBS with 5% BSA. Microscopy slides were prepared by placing a 9 mm diameter Grace Bio-Labs (Bend, OR, USA) SecureSeal imaging spacer, adding 9.1 µL of the labeled cell solution and sealing it with a coverslip. PBMC specimens, prepared as above, were analyzed by high-contrast darkfield and fluorescent microscopy as described [10]. The imaging platform is oil- and calibration-free and fully automated to acquire large numbers of fields of view and perform simultaneous quantitative PNP detection and qualitative cell lineaging through multiparameter fluorescence microscopy. X–Y stage movement, autofocusing modules and automated cell lineaging software allow quantification of PNP binding in low-abundance cell populations. On average, counting of bound PNPs considered 309 cells of the same type per sample (minimum: 17 NK-T cells/sample and maximum 1070 T cells/sample). A Poisson distribution was fit to the PNP-binding events on each cell type. The mean of the Poisson distribution (lambda parameter) corresponds to the average level of MUC16-binding to each immune cell type. We used the exact method to compute a 95% CI for Poisson distributions as described [11]. This method produces non-symmetrical confidence intervals. All computations were made in Python using the Scipy library [12].

### Clinical data

Patient information included serum CA125, complete blood count (CBC) with differential, chemotherapy regimens, imaging studies, operative reports, and clinical notes. A CBC could be linked to each of the 30-study bloods collected, and a CA125 value could be associated on the same day for 17 of the samples. The CBC data included: hematocrit, counts of platelets neutrophils, monocytes, eosinophils, basophils, and three constructed ratios (neutrophil-to-lymphocyte (NLR), platelet-to-lymphocyte (PLR), and lymphocyte-to-monocyte (LMR)). We calculated a Spearman correlation matrix to assess the relationships between average levels of MUC16-binding to each PBMC cell type (B cells, monocytes, NK cells, T cells, and NK-T cells) and serum CA125, and differential counts and ratios. This analysis was performed in SAS v9.4 and all p-values are two-sided.

## Results

**Fig. 1:**
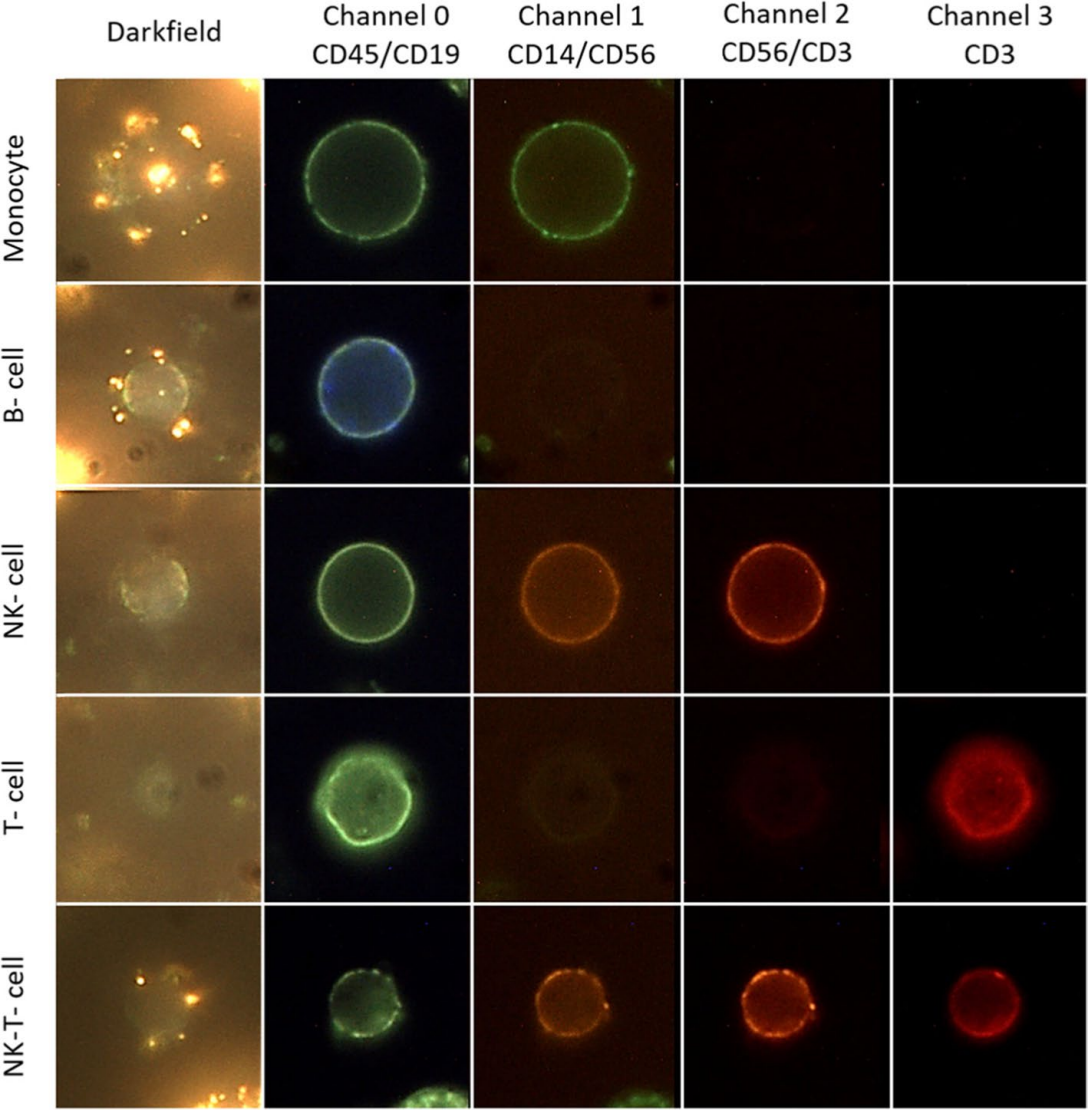
Composite image showing darkfield maximum intensity projection and 4 channel fluorescence of different cell lineages. Legend: Monocytes exhibit high PNP-binding, the B cell medium PNP-binding, and other lineages little or no binding. Fluorophore conjugated anti-CD 45 antibodies were applied as a pan-leukocyte marker visible as green in channel 0. Monocytes were identified with fluorophore conjugated anti-CD14 seen as green on channel 1. B cells were identified using fluorophore conjugated anti-CD 19, visible as blue in channel 0. NK cells with fluorophore conjugated anti-CD56, shows as green at channel 0 and orange in channel 1 and channel 2. T cells were identified using fluorophore conjugated anti-CD3, which appear as red in channel 3. Finally, NK-T cells were identified as green in channel 0, orange in channel 1 and 2 and red in channel 3

Figure 1 is a composite image showing examples of the darkfield-intensity projection of bound PNPs and 4-channel fluorescence of cells with different lineage. We applied fluorophore conjugated anti-CD 45 antibodies as a pan-leukocyte marker, visible as green in channel 0. Monocytes were identified with anti-CD14, seen as green on channel 1. B cells were identified using fluorophore conjugated anti-CD 19, visible as blue in channel 0. NK cells with anti-CD56, shows as green at channel 0 and orange in channel 1 and channel 2. T cells were identified using anti-CD3, which appear as red in channel 3. Finally, NK-T cells were identified as green in channel 0, orange in channel 1 and 2 and red in channel 3. In this example, monocytes exhibit high CA125 binding, the B cell medium CA125 binding, and other lineages little or no binding.

**Fig. 2:**
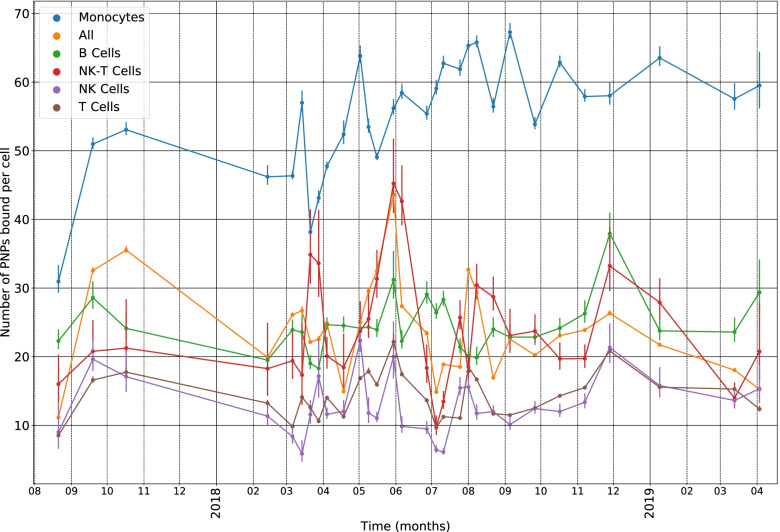
Average PNP-binding per cell to monocytes, B, NK, NK-T, and T cells during 8/2017 to 4/2019

Figure 2 shows the average numbers of PNPs bound to B cells, NK cells, NK-T cells, T cells, and monocytes over the period, 8/2017 to 4/2019, equating with average levels of MUC16-binding to each cell type. Monocytes had the greatest levels of PNP-binding over the period with counts ranging from about 30 to 65 per cell. Cell types with the smallest levels of binding were NK and T cells with the average PNP-binding ranging between 5–20 per cell.

**Fig. 3:**
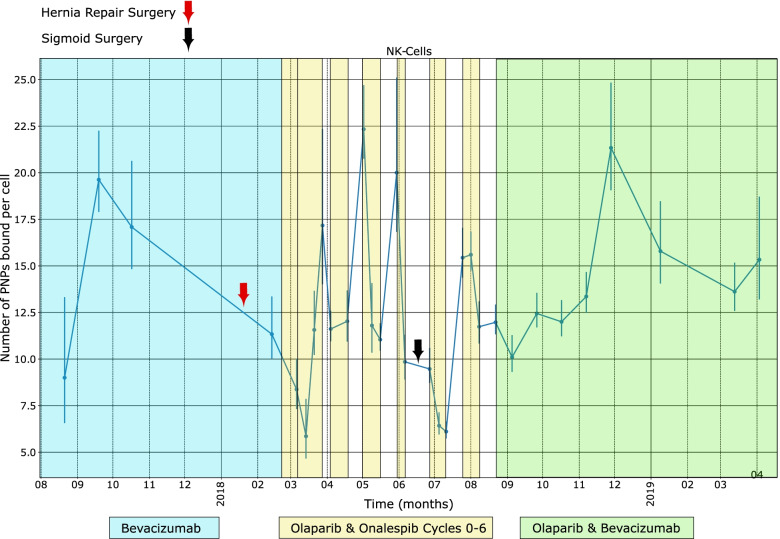
Average PNP-binding to NK cells at time points of clinical evets during 8/2017 to 4/2019. Legend: A rise in NK cell PNP-binding started after beginning Bevacizumab in 10/2017 and resuming it in 12/2018. The patient had two abdominal surgeries indicated by the green and red arrows. The start and end points of the Onalespib/Olaparib cycles are indicated in the yellow background section with each cycle beginning with I.V. administrations of Onalespib

Fluctuations are seen in MUC16-binding over time with each immune cell type. Potential linkage of these fluctuations with clinical events is illustrated in Fig. 3 for NK cells. Peaks in MUC16-binding to NK cells are seen around 10/2017 and 12/2018—both occurring about 2 months after beginning (or resuming) therapy with bevacizumab. During the time-period, 3/2018 to 9/2018, the patient was receiving oral Olaparib continuously and infusions of the investigational heat shock protein-90 (HSP-90) inhibitor, Olanespib. Except for the cycle beginning early April 2018, sharp declines in the level of MUC16-binding to NK cells were seen on the first day of the infusion cycle, with rebounds occurring about 14 days after the infusion and before the next infusion. Finally, the patient had two surgeries in 2018—a hernia repair and sigmoidectomy for residual disease. Both surgeries were followed by a drop in MUC16-binding to NK cells.

**Fig. 4:**
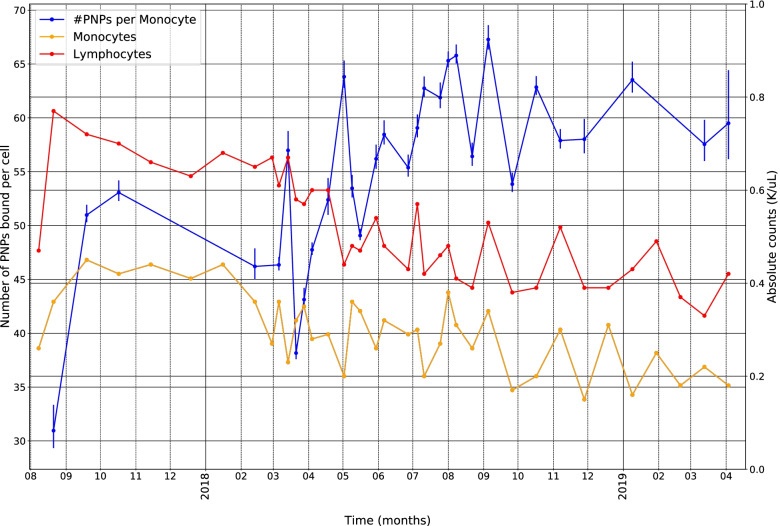
Average PNP-binding to monocytes during 8/2017 to 4/2019 with corresponding blood monocyte and lymphocyte counts

Table 1 highlights significant correlations among the mean levels of MUC16-binding to the immune cell subsets, serum CA125, differential blood counts, and the 3 calculated ratios –PLR, NLR, and LMR. Serum CA125 was positively correlated with averages of MUC16-binding to monocytes (*r* = 0.58, *p* = 0.015), with NLR (*r* = 0.76, *p* = 0.001), with PLR (*r *= 0.67, *p* = 0.003), and inversely correlated with hematocrit (*r* = -0.54, *p* = 0.026). Besides their correlation with CA125, mean levels of MUC16-binding to monocytes were inversely correlated with circulating monocytes (*r* = -0.44, *p* = 0.015) and lymphocytes (*r* = -0.57, *p* = 0.001) and positively correlated with NLR (*r* = 0.48, *p* = 0.007) and PLR (*r* = 0.62, < 0.001). Longitudinal data illustrating the inverse correlations between levels of MUC16-binding to monocytes and circulating monocytes and lymphocytes are illustrated in Fig. 4.

**Table 1:** Significant correlations among levels of MUC-16 binding to PBMC cell types, CA125, and blood count

	**Statistic**	**B-cell mean**	**Mono- cyte** **mean**	**NK-cell mean**	**T-cell mean**	**NK-T cell mean**	**Neutro-phils**	**Mono-cytes**	**Lympho-cytes**	**Eosino-phils**	**Baso-phils**	**NLR**	**PLR**	**LMR**	**Hemato-crit**	**Platelet**
**CA125**	**r**	NS	0.58	NS	NS	NS	NS	NS	NS	NS	NS	0.76	0.67	NS	-0.54	NS
	**p**		0.015									0.001	0.003		0.026	
	**n**		17									17	17		17	
**B-cell mean**	**r**		NS	NS	NS	NS	NS	NS	NS	NS	NS	NS	NS	NS	NS	NS
	**p**															
	**n**															
**Monocyte mean**	**r**			NS	NS	NS	NS	-0.44	-0.57	NS	NS	0.48	0.62	NS	NS	NS
	**p**							0.015	0.001			0.007	0.000			
	**n**							30	30			30	30			
**NK-cell mean**	**r**				0.49	0.43	NS	NS	NS	NS	NS	NS	NS	NS	NS	-0.54
	**p**				0.005	0.017										0.002
	**n**				30	30										30
**T-cell mean**	**r**					0.39	NS	NS	NS	NS	NS	NS	NS	NS	NS	-0.39
	**p**					0.032										0.035
	**n**					30										30
**NK-T cell mean**	**r**						NS	NS	NS	-0.47	NS	NS	NS	NS	NS	-0.54
	**p**									0.008						0.002
	**n**									30						30

*Abbreviations*: *NLR* neutrophil-to-lymphocyte ratio, *PLR* platelet-to-lymphocyte ratio, *LMR* lymphocyte-to-monocyte ratio, *r*   Spearman correlation coefficient, *p*   p-value, *n*   number of data points, *NS*  not significant

Also in Table 1, levels of MUC16-binding to NK cells were positively correlated with levels of MUC16-binding to both T cells (*r* = 0.49, *p* = 0.005) and NK-T cells (*r* = 0.43, *p* = 0.017) and inversely correlated with platelet counts (*r* = -0.54, *p* = 0.002). Levels of MUC16-binding  to T cells were positively correlated with levels of MUC16-binding to NK-T cells (*r* = 0.39, *p* = 0.032) and inversely correlated with platelet count (*r *= -0.39, *p *= 0.035). Finally, Levels of MUC-16 binding to NK-T cells were inversely correlated with eosinophil (*r *= -0.47, *p* = 0.008) and platelet counts (*r *= -0.54, *p* = 0.002). No significant correlations were noted with levels of MUC16-binding to B cells.

## Discussion

Longitudinally-collected and cryopreserved PBMCs from a patient with high grade serous ovarian cancer during a 21-month period of her clinical care were studied using an instrument that combines fluorescent and darkfield microscopy to quantify the amount of MUC16 binding to T cells, B cells, NK cells, NK-T cells, and monocytes. Patterns of binding were then correlated with clinical data. Our discussion will highlight some of the correlations we think are most important.

Figure 2 illustrates considerable variation over time in levels of MUC16-binding by cell type, which, nevertheless, showed good consistency with respect to how the cells ranked in their level of binding. Thus, levels of MUC16-binding to monocytes were greatest at all time points followed by B cells, NK-T, and NK cells and T cells with the lowest levels. This ranking, in the level of binding by cell type in longitudinal data from a single patient, matches the order in the degree of binding in the individual cases reported previously [10]. The ranking may be explained by the degree of expression on white cell surfaces of MUC16’s principal binding partner, Siglec-9 (sialic acid binding Ig-like lectin). Siglec-9 expression among leukocytes has been found to be highest on monocytes, intermediate on neutrophils, and weaker on lymphocytes, including NK and T cells [13] Thus, degree of Siglec-9 expression on leukocytes appears to explain differences in the degree of MUC-16 binding. MUC16-binding to neutrophils also occurs but was not studied here since these are removed during cryopreservation of PBMCs. Although the relative order of binding by cell type was the same between the current and prior study, the case in the current study had levels of binding that were higher for each of the cell types than those seen for the cases in our prior report [10]. This is likely due to the higher ratio of PNPs to cells used to perform gold staining in the set of specimens used in the current study.

We selected NK cells to correlate with details of the patient’s clinical course because of the previous work describing MUC16 inhibition of synapse formation between NK cells and ovarian cancer tumor cells, thus preventing NK-cell tumoricidal function [5–7]. One of the more interesting aspects of Fig. 3 were the troughs and rebounds in MUC16-binding to the NK cells that were seen during February and August 2018, and appeared, in most instances, to coincide with the infusions of the HSP-90 inhibitor, olanespib. Anti-HSP90 treatment is known to modify expression of activating receptors on NK cells [14]. although no specific effect of HSP-90 inhibitors on Siglec-9 receptors has been described. Also, of potential interest, peaks in MUC16 binding to NK cells were seen around 10/2017 and 12/2018—both occurred about 2 months after beginning (or resuming) therapy with bevacizumab. An elevation of NK cells has been described in a clinical trial involving bevacizumab and renal cell carcinoma [15]. Finally, a drop in MUC16-binding to NK cells was seen after the patient’s two surgeries. Abdominal surgery appears to lower NK cell count both experimentally [16] and after primary surgery for ovarian cancer [17]. However, we should note that, in all three of these reports, the increase [15] or decreases [16, 17] relate to NK cell counts and not directly to MUC16- binding to NK cells which was the focus of this report.

Although the clinical correlations noted above must be viewed as descriptive, significance can be attached to the correlations pointed out in Table 1. The only correlation between serum CA125 and binding of MUC16 to the PBMC subtypes was a positive one with MUC16-binding to monocytes (*r* = 0.58, *p* = 0.01). This likely reflects the higher level of Siglec-9 expression on monocytes [13]. The fact that no correlation was seen between serum CA125 and monocyte binding among individual cases in the prior study [10] suggests that there are greater interpersonal than intrapersonal factors that affect the correlation between surface bound MUC16 and serum CA125. In our study, serum CA125 also correlated with the leukocyte ratios, NLR and PLR. Both of the latter correlations have been previously described in women with ovarian cancer using cross-sectional data [18], but we believe this is the first report that the correlations also exist in longitudinal specimens from the same patient. Indeed, the correlation between serum CA125 and NLR of *r *= 0.76, *p* = 0.001 was surprisingly strong based on only 17 observations. CA125 was also weakly and inversely correlated with the patient’s hematocrit likely suggesting chemotherapy-associated anemia, especially with Olaparib, which the patient was on for much of this period [19]. In addition, anemia occurs as a consequence of low erythropoietin response in patients with solid tumors that is, to some extent, independent of chemotherapy [20].

The inverse correlations between monocyte binding and circulating monocyte and lymphocyte counts illustrated in Fig. 3 are also of interest. After entering the blood stream from the bone marrow, monocytes usually infiltrate a wide variety of tissues and become dendritic cells or macrophages [21]. Some also return to the lymph nodes carrying antigens they were previously exposed to, keeping their monocyte form [22]. In either case, these events could lead to a decline in circulating monocytes if MUC16-binding to monocytes promoted macrophage differentiation and/or migration to lymph nodes or other sites of cancer metastases. Increasing expression of the Siglec-9 (or Siglec-7) receptor on mononuclear cells with cancer likely underlies either their differentiation or migration to the tumor or metastatic sites. It has been demonstrated that pancreatic tumors, through the expression of sialic acids on their cell surface engage Siglec-7 and Siglec-9 to mediate monocyte to macrophage differentiation [23]. Additionally, another study demonstrated that Siglec-9 expression is upregulated on tumor infiltrating T cells in lung, colorectal and ovarian cancers [24]. An explanation for the inverse correlation of MUC16-binding to monocytes with circulating lymphocytes is less clear; but macrophage plasticity and interaction with lymphocytes subsets may be relevant [25].

Similar to findings among individual cases in the prior study [10], we saw positive correlations between levels of MUC16-binding to NK cells and T cells as well as levels of MUC16-binding to NK-T cells and T cells. This may reflect overlapping of markers for distinguishing these subsets. In our study, levels of MUC16-binding to all three of these subsets was inversely correlated with the platelet count. This is of interest because activated platelets enhance T cell, cytotoxic T cell, NK cell, and B cell infiltration of tissue and subsequent migration to sites of tissue inflammation, such as occurs with solid tumors. Better understanding of platelet-lymphocyte “cross-talk” as described by Li [26] and how this affects MUC16-binding awaits the development of routine methods for specifically identifying and counting activated platelets.

In conclusion, we have described the application of a novel imaging platform that combines darkfield and fluorescent microscopic techniques to quantify binding of MUC16 to longitudinally collected PBMC subtypes in a patient with EOC. MUC16-binding to monocytes and to NK cells appeared to correlate best with CA125 and CBC data as well as clinical events–if only on a descriptive basis for the latter. Besides illustrating the potential values of the imaging platform in clinical research, we believe this work also establishes the potential research value of longitudinally collected and cryopreserved PBMCs which, we believe, are currently underutilized in cancer research.

## Data Availability

The data that support the findings of this study are available upon request to the corresponding author with appropriate institutional approvals. The data are not publicly available due to privacy and ethical restrictions.
